# The Effect of Lemon Juice (*Citrus limon* L.) Treated with Melatonin on the Health Status and Treatment of K14HPV16 Mice

**DOI:** 10.3390/antiox13050588

**Published:** 2024-05-10

**Authors:** Fátima Badiche-El Hilali, Beatriz Medeiros-Fonseca, Jéssica Silva, Ana C. Silvestre-Ferreira, Maria João Pires, Rui M. Gil da Costa, Francisco Peixoto, Paula A. Oliveira, Daniel Valero

**Affiliations:** 1 Department AgroFood Technology, EPSO-CIAGRO, University Miguel Hernández, Ctra. Beniel km. 3.2, 03312 Orihuela, Spain; 2 Center for the Research and Technology of Agro-Environmental and Biological Sciences (CITAB), University of Trás-os-Montes and Alto Douro (UTAD), 5001-801 Vila Real, Portugal; 3 Center for Animal and Veterinary Science (CECAV), University of Trás-os-Montes and Alto Douro (UTAD), 5001-801 Vila Real, Portugal; 4 Post-Graduate Programme in Adult Health (PPGSAD), Federal University of Maranhão, São Luís 65085-580, Brazil; 5 Health Sciences Center, State University of the Tocantins Region of Maranhão (UEMASUL), Imperatriz 6591-480, Brazil; 6 Chemistry Center-Vila Real (CQ-VR), Biological and Environment Department, School of Life and Environmental Sciences, University of Trás-os-Montes and Alto Douro (UTAD), P.O. Box 1013, 5001-801 Vila Real, Portugal

**Keywords:** lemon, melatonin, valorization, antioxidant, HPV16, human papillomavirus

## Abstract

Lemon is a fruit rich in antioxidant properties and has several health benefits, namely the reduction of skin edema and anticarcinogenic properties, which are due to its high content of bioactive compounds. Melatonin can improve and preserve the properties of lemon for longer and also has health benefits. The aim of this study was to evaluate the effects of oral administration of lemon juice after melatonin treatment on murinometric parameters of wild-type (WT) mice and transgenic mice carrying human papillomavirus (HPV). Two trials were performed for oral administration of the lemon extract compound: in drinking water and in diet. First of all, lemons were treated by immersion with melatonin at 10 mM. Then, lemons were squeezed, and the juice obtained was freeze-dried and stored to be subsequently added to drinking water or diet, according to the assay. Thus, mice were divided into eight groups in the drink assay (each with n = 5): group 1 (G1, WT, control), group 2 (G2, WT, 1 mL lemon), group 3 (G3, WT, 1.5 mL lemon), group 4 (G4, WT, 2 mL lemon), group 5 (G5, HPV16, control), group 6 (G6, HPV16, 1 mL lemon) group 7 (G6, HPV16, 1.5 mL lemon) and group 8 (G6, HPV16, 2 mL lemon). The diet assay was divided into four groups: group 1 (G1, WT, control), group 2 (G2, WT, 4 mL lemon), group 3 (G3, HPV16, control) and group 4 (G4, HPV16, 4 mL lemon). In the drink assay, the highest concentration of melatonin (308 ng/100 mL) was for groups 4 and 8, while in the food assay, there was only one concentration of melatonin (9.96 ng/g) for groups 2 and 4. Both trials lasted 30 days. During this time, body weight, food and water were recorded. Afterward, they were sacrificed, and samples were collected for different analyses. At the concentrations used, the lemon juice with melatonin had no adverse effects on the animals’ health and showed a positive outcome in modifying weight gain and enhancing antioxidant activity in mice. Moreover, a reduction in the incidence of histological lesions was observed in treated animals. Further research is needed to better understand the effects of lemon extract on health and treatment outcomes in this animal model.

## 1. Introduction

Lemon is a citrus fruit widely used throughout the world due to its versatility and unique flavor. In addition to being a rich source of vitamin C, lemons also contain other beneficial compounds, such as citric acid and flavonoids, which may have positive effects on health [1]. Regular consumption of lemon and other vitamin C-rich foods has been associated with a lower incidence of chronic diseases, such as cardiovascular disease, cancer and neurodegenerative diseases [2]. Furthermore, the citric acid present in lemons may help prevent the formation of kidney stones by increasing the excretion of calcium in the urine [3]. However, lemons can suffer from various pathologies that can reduce their shelf life and nutritional quality. For example, rot caused by fungi and bacteria can damage lemons and render them unusable. To address this issue, various preservation techniques have been studied, including the use of post-harvest treatments with melatonin, which has been shown to improve fruit quality and prolong shelf life [4]. Melatonin (MEL), or N-acetyl-5-methoxytryptamine, is an indoleamine that was first discovered in the pineal gland of vertebrates [5] and, five decades later (1995), in the plant kingdom. MEL, as a phytohormone produced naturally in plants, including citrus fruits, has been found to have a role in a wide range of physiological processes in plants [6]. Furthermore, the application of MEL to lemons has been found to reduce the incidence of fungal and bacterial diseases in the fruit, maintaining its nutritional quality and prolonging shelf life [7,8,9]. Also, melatonin has been shown to have antioxidant and anti-inflammatory effects on human health that may help prevent cancer and other HPV-induced diseases [10]. In addition, exposure of SARS-CoV-infected cells to melatonin has been shown to inhibit the growth and proliferation of the virus [11]. The use of melatonin as a potential treatment in animal models has also shown promising results in other studies [12].

On the other hand, in vivo animal studies, particularly those conducted on mice and rats, are an important tool in biomedical research due to the similarities between these animals and humans in terms of genetics, physiology and anatomy [13]. These studies can provide valuable information on the safety and efficacy of treatments, as well as insight into the underlying mechanisms of diseases. The K14HPV16 mouse is a transgenic model of cancer induced by human papillomavirus (HPV). HPV is the most common sexually transmitted infectious agent worldwide [14]. The HPV16 is responsible for the majority of cases of cervical cancer, as well as other anogenital and head and neck cancers [15]. The potential link between HPV infection and lemon consumption has not been extensively studied, but some research has suggested that citrus fruits such as lemon may have anti-cancer properties due to their high levels of flavonoids and other bioactive compounds [16]. However, more research is needed to fully understand the relationship between citrus fruit consumption and HPV-related diseases.

Often, post-harvest studies are carried out on fruits to enhance and extend their useful life. However, it is usually not studied if there is any effect on human health after eating these fruits or foods. Therefore, the aim of this study was to study the effects of lemon with aqueous melatonin extract in an in vivo mouse model to see if there is any effect on the health and well-being of HPV16 pathology mice and wild-type mice. This model was previously used by our team to test other natural compounds, making it a suitable model for testing both the efficacy and possible hepatotoxicity of lemon extracts.

## 2. Materials and Methods

### 2.1. Lemon, Lemon Fruit Juice and the Soluble Extracts

Lemon Verna fruit (*Citrus limon* (L.) Burm. F. var. Verna) was harvested in the summer season of 2022 from a commercial citrus organic orchard in Orihuela (Alicante), Spain. The fruit was immediately transported to the laboratory. Subsequently, the fruit was washed with distilled water. After drying at room temperature, the fruit was treated with melatonin (purchased from Sigma, Sigma-Aldrich, Madrid, Spain) aqueous solution at 10 mM by immersing for 30 min. Samples were freshly squeezed for lemon juice and then lyophilized (FreeZone 4.5, Labconco, Kansas City, MO, USA) in order to preserve their chemical composition as much as possible until they were used. Afterward, the sample was reduced to a doughy dense mass and preserved in a freezer at −20 °C until further analysis. For oral intervention study in mice via drinking water, we used a concentration of freeze-dried lemon juice of 38.6, 57.8 and 77.1 mg/100 mL to obtain a dose equivalent to 1, 1.5 and 2 mL, respectively, of lemon juice reconstituted in the final volume of water administered to the mice. From lower to higher juice concentrations, the melatonin dose was 154, 231 and 308 ng, respectively, based on the concentration of melatonin in this proportion of freeze-dried lemon juice. For the second trial, the diet test, we dissolved 8 g of freeze-dried lemon juice in 100 mL of water and added it to 2 kg of normal food. In other words, the concentration of freeze-dried juice in the food is 4 mg/g, which is equivalent to 4 mL of rehydrated lemon juice, in which we can find 9.96 ng of melatonin.

### 2.2. Phenolic Compounds Profile and Stability of the Aqueous Extract

For identification and quantification of phenolic compounds, we use the extraction described in Gironés-Vilaplana et al. (2012) [17] with slight modifications. Regarding HPLC system, water/formic acid (99:1, *v*/*v*) and acetonitrile were used as the mobile phases A and B, respectively, with a flow rate of 1 mL per min. The injection volume was 20 µL, and chromatograms were recorded at 320 and 360 nm in an Agilent HPLC 1200 Infinity series equipped with a photodiode array detector (Agilent Technologies, Waldbronn, Germany) and a mass detector in series (Bruker Daltonics Ultra HCT-ESI Ion Trap, Bremen, Germany) and a Luna C18 column (250 × 40 mm, 5 µm particle size). The ionization conditions were 350 °C and 4 kV for capillary temperature and voltage, respectively. The nebulizer pressure and nitrogen flow rate were 65.0 psi and 11 L/min, respectively. The full-scan mass covered the range of *m*/*z* from 100 to 1200. Individual phenolics quantification was performed in duplicate in each sample by using an HPLC-DAD system with the same conditions that were used for phenolics identification. Individual phenolic compounds were identified by their mass in an HPLC-DAD-ESI/MS, their spectra and retention time, using previous bibliography [18]. Moreover, some of them were corroborated using analytical standards. For quantitative analysis, a calibration curve of two standards, 5-*O*-caffeoylquinic acid and 3-luteolin-*O*-rutinoside (Sigma Aldrich, Germany), was used for the quantification of hydroxycinnamic acids and luteolin derivatives at 320 and 360 nm, respectively. The total identified polyphenol concentration was calculated as the sum of the individual phenolic concentrations, and the results were expressed in mg per g of lemon juice.

The stability of the rehydrated lemon juice was evaluated for 5 consecutive days at room temperature. In this study, the aqueous extract was prepared at the same concentration that was provided to the mice in drinking water and analyzed daily through a colorimetric analysis described in another study [19] to visualize if there was degradation of ascorbic acid in the lemon juice. On the other hand, total antioxidant activity was measured by the ABTS-peroxidase system and total phenols by the Folin–Ciocalteu method, both previously analyzed in another study on lemon [20].

### 2.3. Mice

Forty female mice were used for the drinking study: twenty transgenic (HPV16+/−) and twenty wild-type (WT) (HPV16−/−), aged 18–20 weeks old. For diet test, only twenty mice were used: 10 WT and 10 HPV16+/−, aged 30 weeks old. Cutaneous lesions in this mouse strain begin to proceed from the hyperplastic to the dysplastic stage at the age of 20–22 weeks [21], creating an opportunity to test new strategies to block this progression. The mouse strain [22] was donated by Drs Jeffrey Arbeit and Douglas Hanahan from the University of California through the National Cancer Institute Mouse Repository (Frederick, MD, USA). The animals were genotyped weaning, using tail tip samples by using a polymerase chain reaction technique previously used in our works.

### 2.4. Experimental Procedures

The experimental procedures were approved by the national authorities (approval number 014139) and carried out at the University of Trás-os-Montes and Alto Douro animal facilities. The animals were kept under controlled experimental conditions. All mice were acclimated for four weeks in a controlled environment (20 ± 2 °C, 12 h light/dark cycle and relative humidity 50 ± 10%) and had free access to food and water. Two experiments were performed. In one of them, we administered freeze-dried lemon through drinking water, and in the other, we administered the preparation through the animals’ diet. The first is called “drink test” or “drink assay”, and the second one is called “diet test” or “food test”. For the diet assay, a commercial rodent feed (certified Mucedola 4RF21, Milan, Italy) was used as the basis for the preparation of modified diets containing melatonin-treated lemon extracts. Lemons that were lyophilized were reconstituted with water to form a juice and incorporated into a modified diet at concentrations of 0.4% (*w*/*w*). These concentrations were calculated considering the maximum daily recommendations for melatonin (5 mg/d) for an adult (70 kg). For a 30 g mouse with an average daily intake of 5 g, this corresponds to 19.93 µg. Diets were prepared using an industrial mixer (CPM Europe, model C-300, Zaandam, The Netherlands) and adding 5% (*w*/*w*) water to the mixture to form new pellets (4.2 mm diameter). The base diet was prepared following the same method but without the addition of lemon extract. Subsequently, all batches of feed were dried in an oven at 40 °C for 48 h and stored at 4 °C until the feed was ready for use. Throughout the first experiment (drink assay), mice were fed a standard diet (4RF21 GLP, Mucedola, Italy) ad libitum. For the drink assay, animals were divided into eight groups (each with n = 5). Groups 1 to 4 were wild-type (WT), and groups 5 to 8 were transgenic (HPV16): group 1 (G1, WT, control), group 2 (G2, WT, 1 mL lemon), group 3 (G3, WT, 1.5 mL lemon), group 4 (G4, WT, 2 mL lemon), group 5 (G5, HPV16, control), group 6 (G6, HPV16, 1 mL lemon) group 7 (G7, HPV16, 1.5 mL lemon) and group 8 (G8, HPV16, 2 mL lemon). For the diet assay, mice were divided into four groups of 5 animals each: group 1 (G1, WT, control), group 2 (G2, WT, 4 mL lemon), group 3 (G3, HPV16, control) and group 4 (G4, HPV16, 4 mL lemon). Animals’ body weight, water and food intake were recorded and monitored every 5 days and were known as “sample dates”. At the same time, animals were carefully observed to confirm their well-being through their humane endpoint evaluation. The lemon juice melatonin-enriched extract was administered in drinking water for 30 days at different concentrations and was renewed every 48 h. The diet test also lasted 30 days, and the animal’s diet food was added every 72 h. At the end of the 30 days, all animals (both experiments) were sacrificed by intraperitoneal administration of a mixture of xylazine and ketamine, followed by cardiac puncture exsanguination, according to FELASA guidelines, and biological samples of blood and organs (heart, lung, liver, spleen, kidneys, as well as chest and ear skin samples) were collected for analysis.

#### 2.4.1. Determination of Microhematocrit and Total Plasma Proteins (TPP)

The hematocrit was measured using microhematocrit method. Samples were centrifuged at 9000 rpm for 5 min, and the height of the packed red blood cells was measured with a graduated ruler. The results were expressed as the percentage of blood cell volume. For the serum biochemistry, blood samples were allowed to clot and centrifuged at 3000 rpm for 15 min (4° C). To measure the total plasma proteins, the blood samples were allowed to clot and centrifuged at 3000 rpm for 15 min (4° C). The serum concentrations of TPP were determined in an autoanalyzer (Prestige 24i, Cormay PZ, Marynin, Poland).

#### 2.4.2. Histological Analysis

Samples of heart, liver, kidney, lung and spleen were collected and immediately fixed in 10% neutral buffered formalin for at least 24 h. The fixed tissues were then dehydrated through a graded series of ethanol, cleared with xylene and embedded in paraffin wax. The tissue sections were stained with hematoxylin and eosin (H&E) to evaluate the histology of the organs. The H&E staining method is commonly used to visualize the general architecture and cellular details of tissue sections. The stained sections were examined under a light microscope by a trained histopathologist. The evaluation criteria included the presence of inflammation, fibrosis, necrosis, cellular infiltrates and any other histopathological changes that may indicate tissue damage or disease. Lesions were classified as previously described for this mouse strain [23]. The histopathological analysis was performed in a blinded manner, where the histopathologist was unaware of the treatment groups or experimental conditions. The results of the histopathological analysis were recorded and used to draw conclusions about the effects of the lemon juice treatment on the organs.

#### 2.4.3. Hepatic and Kidney Oxidative Stress

The levels of oxidative stress markers were measured in liver and kidney tissue homogenates. Both organs were homogenized in cold buffer solution (0.32 mM of sucrose, 20 mM of HEPES, 1 mM of MgCl_2_ and 0.5 mM of phenylmethyl sulfonyl fluoride PMSF, prepared in ethanol to prevent protein degradation, pH 7.4) using a motor-driven Teflon and glass Potter-Elvehjem homogenizer. The homogenate was centrifuged at 10,000 rpm for 20 min at 4 °C (Sigma model 3K30, Osterode, Germany), and supernatants were collected for analysis. Superoxide dismutase activity (Cu/Zn-SOD) was determined by the nitroblue tetrazolium (NBT) reduction generated by superoxide radicals generated by xanthine oxidase system at 560 nm [24]. For quantitative analysis, a calibration standard curve constructed by SOD from bovine erythrocytes was used (0–3.75 U mL^−1^). The activity of catalase (CAT) was determined at 240 nm in accordance with a previously published method [25] and was calculated using bovine catalase as a standard (0–5 U mL^−1^).

### 2.5. Statistical Analysis

Statistical analysis was performed using the SPSS program (Statistical Package for Social Sciences, Chicago, IL, USA) version 17. A statistical ANOVA followed by the Bonferroni test was performed, and values of *p* < 0.05 were considered statistically significant.

## 3. Results

### 3.1. Phenolic Compounds Profile and Stability of the Extract

Table 1 summarizes the chemical composition analyzed present in lemon juice. Ten compounds were detected, the two major ones being hesperidin and eriocitrin (flavanones). On the other hand, some compounds, such as luteolin-7-*O*-rutinoside (flavone) or quercetin 3-*O*-glucoside (flavanol), showed a very low concentration. Gallic acid and cynarin were not detected. Hesperidin was the most abundant compound in the lemon juice, with 69.9 ± 3.9 mg 100 mL^−1^, followed by eriotricin (19.5 ± 0.6 mg 100 mL^−1^). On the other hand, diosmetin 6,8-di-*C*-glucoside and diosmetin 8-*C*-glucoside were the next most important compounds present in lemon juice (13.7 ± 0.2 and 12.9 ± 0.3 mg 100 mL^−1^, respectively). Otherwise, the compounds with the lowest concentration in the juice were quercetin 3-*O*-glucoside, caffeic acid (hydroxycinnamic acid) and luteolin-7-*O*-glucoside with an amount of 1.2 ± 0.0, 1.5 ± 0.0 and 1.5 ± 0.2 mg 100 mL^−1^, respectively. In terms of the stability of the aqueous extract resulting from redissolving the freeze-dried lemon material in water, it was studied for 96 consecutive hours (4 days) at room temperature, and it was visualized that by day 3 or 72 h, the concentration of phenolic compounds began to decrease (Table 2) in terms of vitamin C and total antioxidant activity. Therefore, the feeding water was maintained up to a maximum of 72 h to avoid degradation of these compounds. At 96 h, significant differences were observed in all parameters compared to 72 h. Vitamin C content remained around 30 mg 100 mL^−1^ until 96 h when it decreased to 22.3 ± 1.6 mg 100 mL^−1^ (*p* < 0.05). As for the content of total phenolic compounds and total antioxidant activity at 96 h, a decrease of 7 and 6 mg 100 g^−1^, respectively, was observed in relation to the content at 72 h (*p* < 0.05).

### 3.2. Mice Experiments

**Figure 1:**
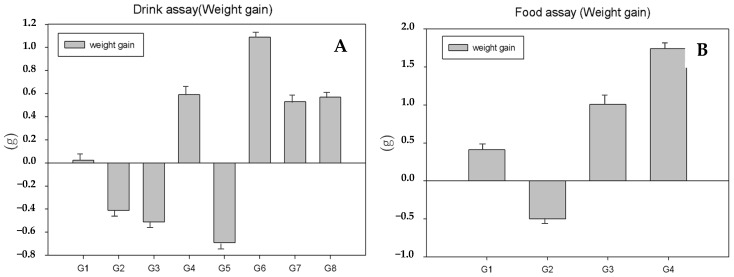
The weight gain in grams (mean ± SD) in drink assay (**A**) for each group ((G1, WT, control), (G2, WT, 1 mL lemon), (G3, WT, 1.5 mL lemon), (G4, WT, 2 mL), (G5, HPV16, control), (G6, HPV16, 1 mL lemon), (G6, HPV16, 1.5 mL lemon) and (G6, HPV16, 2 mL lemon)) and food assay (**B**) for each group ((G1, WT, control), (G2, WT, 4 mL lemon), (G3, HPV 16, control) and (G4, HPV 16, 4 mL lemon)).

During the experimental work, the animals showed no signs of behavioral change, nor did we register mortality. Table 3 and Table 4 show the animal’s body weight variation in both experiments for the different groups under study. In the drink assay (Table 3), we can see a decrease in body weight in groups 2, 3 and 5. Over the sample dates, the values of body weight show statistically significant differences between groups. In the second sample date, we can observe statistically significant differences between wild-type groups 1 and 2 (*p* < 0.05); also, group 2 was statistically different from groups 3 and 4 (*p* < 0.05). In the fourth sample date, the statistically significant differences were presented between group 5 and group 6 (*p* < 0.05) and group 7 and group 8 (*p* < 0.05). There was also a statistically significant difference in the sixth sample date between group 3 and group 2 (*p* < 0.05). Regarding the average weight gain (Figure 1A), the results showed statistically significant differences between the control and WT groups 2 and 3 (*p* < 0.05) and also between the control HPV16 (G5) and HPV16 treated groups (G6, G7 and G8) (*p* < 0.05). In the diet test (Table 2), at the beginning of the trial, the average body weight was between 26.96 and 29.87 g; at the end, these values were 30.28 and 28.45 g. Significant differences were not found between the animal’s weight at the beginning and at the end of the trial. However, the consumption of lemon in group 2 was lower, and there was a lower weight than group 1 during the whole trial. Regarding mean weight gain (Figure 1B), statistically significant differences were found between groups 2 and 4 (*p* < 0.05)—these two groups were exposed to lemon extract.

**Figure 2:**
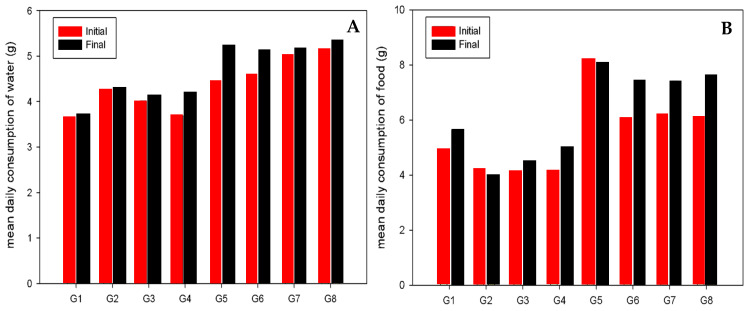
Mean daily consumption (gram) of water (**A**) and food (**B**) per animal in each group ((G1, WT, control), (G2, WT, 1ml lemon), (G3, WT, 1.5 mL lemon), (G4, WT, 2 mL lemon), (G5, HPV16, control), (G6, HPV16, 1 mL lemon), (G6, HPV16, 1.5 mL lemon) and (G6, HPV16, 2 mL lemon)) at the beginning and at the end of drink test.

**Figure 3:**
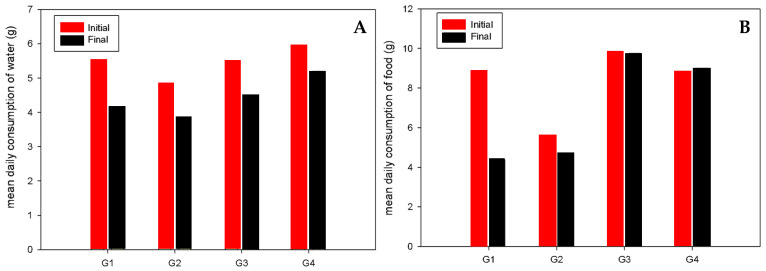
Mean daily consumption (gram) of water (**A**) and food (**B**) per animal in each group ((G1, WT, control), (G2, WT, 4 mL lemon), (G3, HPV 16, control) and (G4, HPV 16, 4 mL lemon)) at the beginning and at the end of diet test. Relative organ weights (grams) of the drinking test (mean ± standard error) and in food assay.

The average food and water consumption for the water administration test is represented in Figure 2. We can observe that the transgenic groups (G5, G6, G7 and G8) have a higher consumption of both water and food at the end of the trial. Moreover, in the diet test, the group with the highest water consumption was group 4, as can be seen in Figure 3A. For food consumption, group 1 (WT) had the lowest intake at the end of the trial, and group 5 (HPV16) had the highest intake at both the start and end of the test with an average of 9.75 g per animal at the end of the test (Figure 3B). In general, the groups composed of transgenic animals had higher consumption than wild-type animals.

In the drink test, relative organ weights (Table 5) showed significative differences between group 1 and group 5 in the spleen and heart (*p* < 0.05), being that these organs were heaviest in the transgenic group in comparison with the WT group. Also, the liver of WT group 3 presented a smaller size than the liver of HPV16 group 7. Concerning other organ weights, there were no significant differences. In Table 6, the relative organ weights of the food test were represented. We can observe that there were no significant differences between any of the groups.

### 3.3. Microhematocrit and TPP Values

From Table 7 and Table 8, we can observe the microhematocrit and the total plasma protein (PPT) value parameters for the different groups under study for both experiments. In the drink test, wild-type mice show slightly elevated microhematocrit compared with HPV16, in contrast to the PPT value, which is mildly higher in HPV16 compared with wild types. This difference did not reach statistical significance. Moreover, between the treated and untreated mice (wild-type as well as HPV 16), there were no significant differences (Table 7). In the food test, we observed similar results (Table 8).

### 3.4. Histology

**Figure 4:**
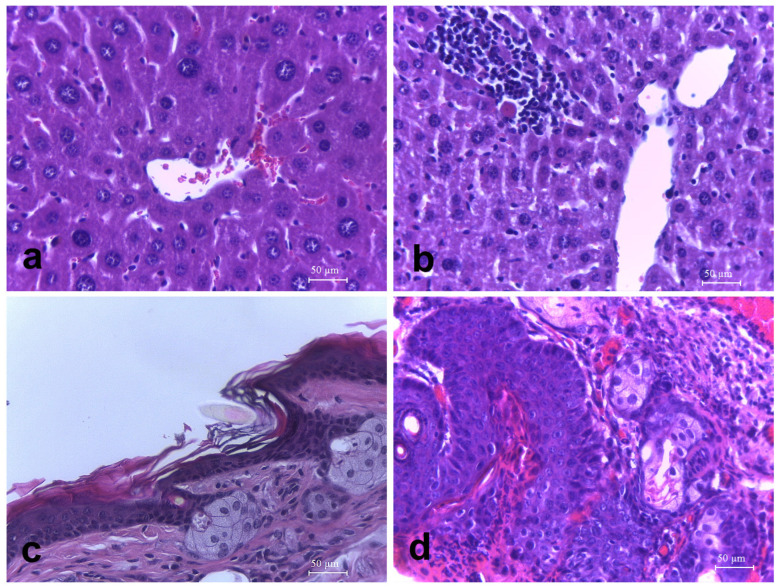
Representative histological images (hematoxylin and eosin, 400×). (**a**) Normal hepatic histology, group 5 mouse (HPV16, control). (**b**) Focal leukocytic infiltration, midzonal to centrilobular, with hepatocellular necrosis, group 4 mouse (WT, 2 mL lemon). (**c**) Normal skin histology, group 1 mouse (WT, control). (**d**) Epidermal dysplasia, group 5 mouse (HPV 16, control).

The results of the heart, lung, liver, spleen and kidney histology tests for the drink test are summarized in Table 9 and Figure 4. There were no significant differences in histological parameters. Transgenic animals treated with lemon juice (G6, G7, G8) showed fewer kidney lesions compared with the control group (G5). The results about chest skin in the “normal” parameter showed that group 1 is significantly higher than group 5 (*p* < 0.05), group 3 is significantly higher than group 7 (*p* < 0.05), and group 4 is statistically higher than group 8 (*p* < 0.05). The results about the “normal” parameter in ear skin showed that group 1 is significantly higher than group 5 (*p* < 0.05), as well as group 3 with respect to group 7 (*p* < 0.05) or group 4 with respect to group 8 (*p* < 0.05). For the diet test (Table 10), there were significant differences only between group 1 and group 3 (*p* < 0.05) and group 2 and group 4 (*p* < 0.05) in the “normal” parameter of both chest and ear skin.

### 3.5. Oxidative Stress

Concerning hepatic oxidative stress analyses, significant differences were observed between groups for two of the markers included in this study. The results of the drink test presented in Table 11 showed that wild-type (WT) treated groups (G2, G3 and G4), when compared with the wild-type control group (G1), have a slight increase in all enzymatic activities (*p* < 0.05). Thus, there were significant differences between groups 3 and 4 and group 1 (*p* < 0.05) in SOD activity. Moreover, there were also significant differences between the WT treated groups (*p* < 0.05), being that group 4 was the one with the highest amount of SOD (42.87 ± 7.67 U min^−1^mg^−1^). In CAT activity, G4 presented significant differences between untreated WT (G1) and treated groups 2 and 3. On the other hand, HPV16 groups also showed significant differences between treated (G6 and G7) and control group (G5) (*p* < 0.05), both in SOD and CAT activity. Group 6 presented the lowest activity (SOD: 19.34 ± 4.98 U min^−1^mg^−1^/CAT: 7.65 ± 1.03 mmol H_2_O_2_ min^−1^mg^−1^)). In the diet test, no significant differences were observed between groups for any of the enzymatic activity markers (Table 12).

## 4. Discussion

Traditionally, fruits and vegetables have been used for medicinal purposes, providing the treatment of various diseases. These foods have contributed greatly to the development of new therapeutic strategies thanks to their secondary metabolites or bioactive compounds, which interact with cellular targets. Lemon contains numerous beneficial bioactive compositions, including phenolic compounds (mainly flavonoids), vitamins, carotenoids, essential oils, minerals and dietary fiber [26] with anti-inflammatory, antimicrobial and antitumor activities [27]. However, this fruit has a reduced shelf life, and one of the ways to maintain these compounds for a longer time is the application of post-harvest treatments such as melatonin, which has been shown to have several effects on quality maintenance [8,9]. The profile of phenolic compounds identified in lemon juice extract presented the flavanones hesperidin and eriocitrin as the predominant compounds above the flavones diosmetin-6,8-di-*C*-glucoside and diosmetin-8-*C*-glucoside, which is consistent with Gonzalez-Molina et al.’s (2009) previous studies [26] in which the most abundant compounds were hesperidin and eriocitrin, although with the difference that, in this study, the most abundant compound was eriocitrin instead of hesperidin, and the predominance was not in equal proportion (3:6 vs. 1:5). Hesperidin accounts for almost 50% of the total phenolic compounds in our lemon phenolic compounds’ composition, as reported other authors [28]. Hesperidin has shown numerous positive effects on human health; however, in this work, one of the most important effects is restricting virus replication and progression [29,30]. Other compounds found with a considerable content were scoparin and vicenin-2 (12 ± 0.5 and 11.5 ± 0.2 mg g-1, respectively). This content was higher than that found in other works, as well as the total content of phenolic compounds [26,31]. We can also highlight the presence of other compounds in very low concentrations (luteolin-7-O-rutinoside, quercetin-3-O-glucoside, caffeic acid or rutin) but which may have an important and significant role in decreasing intracellular ROS concentration and in protecting lipid, DNA and mitochondrial functionality from the damage induced by free radicals [32]. Other works [28,33] show different profiles of polyphenolic compounds from ours. This occurs, for example, in the work of Gonzalez-Molina et al., 2009 [26], in which they mention the presence of diosmin among the most notable compounds in lemon Verna, while in our work, we did not find this compound. This could be due to factors such as the variety, the ripening stage at which the fruit is harvested, the water content or the part of the fruit analyzed, as well as the type of analysis carried out.

The observation of a decrease in the concentration of phenolic compounds, as well as vitamin C and total antioxidant activity, over 72 h of storage, is consistent with studies demonstrating the sensitivity of these compounds to adverse environmental conditions [34]. These findings highlight the importance of considering appropriate preservation strategies to maintain the stability of bioactive compounds in lemon juice during storage and processing [35].

To reach the proposed goals, two experimental tests were carried out in parallel to assess how the consumption of melatonin-treated lemon affects both healthy (WT) and sick (HPV16) animals. It is known that in experimental laboratory work, it is important to observe the animals regarding their well-being, and whenever there is discomfort on the part of the animals, they must be sacrificed in advance [36]. However, the humane endpoints evaluated and recorded weekly never reached the sum of 4, a value from which the animals would have to be sacrificed before the scheduled date for the end of the test. It is therefore concluded that, in accordance with our results, the exposure of animals to *C. limon* juice treated with melatonin appears to be safe. We can, therefore, conclude that fruits treated with compounds such as melatonin (which is a natural origin elicitor) at these doses do not have any toxicity for animals and humans, as has already been proven in other studies [37], so it is advisable to consume them in the doses studied, which extrapolated to a human with an average weight of 70 kg would correspond to 5 l of lemon juice.

In the drinking test, fluctuations in body weight and weight gain of mice for the different groups were observed throughout the study, with significant differences between groups. These differences in body weight may indicate possible effects of lemon juice with melatonin on the metabolism and physiology of the mice, as were found in other research in which the chemical components present in lemon, such as hesperidin, may have effects on metabolism and physiology [28,38]. In addition, Saini R.K. et al. (2022) support the idea that lemon juice may affect the physiology of mice [28]. In the diet test, although no significant differences in body weight were observed between the beginning and end of the study in either group, differences in mean weight gain were observed between the groups exposed to lemon juice with melatonin. The lemon extract did not cause dose-dependent changes in body weight or weight gain. However, it is worth noting that untreated HPV16-transgenic mice showed weight loss during the experimental period, while all transgenic mice treated with lemon extract showed mild weight gains. These findings suggest that the administration of melatonin-treated lemon may have different effects on weight gain in WT and HPV16 animals, showing that healthy animals tend to lose weight while sick animals tend to gain weight. These statistical differences support the hypothesis that the extract is safe under these experimental conditions and may have a favorable impact on the animal’s health status. The average water consumption for each group was constant throughout the tests, which agrees with the results published in a study that evaluated the safety of green tea ingestion in ICR mice, where it was concluded that the average consumption of both food and drink did not vary regardless of tea concentration [39]. These results also imply that the extract was palatable enough not to impair the animal’s drinking behavior, which was a limitation in our study because the administration of lemon juice in other studies is commonly carried out by intragastric administration or via oral gavage [40,41]. In addition, it was also found that in both tests, the transgenic animals showed a higher consumption of water. This observation is reported in other studies and explained by the fact that transgenic animals develop skin lesions, thus losing their barrier functions in controlling hydration and, therefore, they need to ingest more water in order to reach balance [42]. Concerning the relative masses of organs, no statistical differences were found between treated and untreated groups in both experiments; however, some differences were found between the WT and HPV16 groups, which is consistent with other studies [43]. In agreement with the observations in water consumption, transgenic mice showed higher concentrations of total plasmatic proteins in blood samples, suggesting mild dehydration. The hematocrit was lower in HPV16 animals compared with WT mice, and the lemon extract improved the hematocrit values, further suggesting it may have a positive effect on these animals’ health. Furthermore, histological analyses did not reveal lesions associated with the extract. Transgenic animals showed hepatic lesions typical of their strain, regardless of the treatment in accordance with other works [44] although it should be noted that the lemon extract showed fewer kidney lesions in HPV16 treated groups compared to the control. Antioxidant enzymes, such as SOD and CAT, represent the defense response system to excess ROS. In our study, treatment with lemon juice aqueous extract significantly decreased SOD and CAT activity in HPV-16 groups. The increased SOD activity reflects the possible activation of a compensatory mechanism to counteract free radicals in the liver, so lemon juice treatment in HPV16 prevented ROS accumulation by decreasing SOD and CA activity. These results were similar to other studies [40], showing that the extract has a favorable toxicological profile. The liver of K14HPV16 mice is particularly prone to inflammation, and therefore, this animal model is useful to test the potential hepatotoxicity of natural compounds.

## 5. Conclusions

The administration of *Citrus limon* (L.) Burm. F. var. Verna treated with melatonin had no negative effects on the welfare of the animals both when it was administered in the drinking water and when it was administered in the food, so we can conclude that the consumption of this compound, under these circumstances, is not toxic. Lemon juice showed positive results in modifying weight gain, which means that it can have an effect on the metabolism and physiology of wild-type and transgenic mice. It was observed that the transgenic animals that were exposed to lemon extract in both tests had a higher water consumption than the other animals, which is not related to the extract consumption. The animals treated with lemon extract showed a trend toward a reduction in the incidence of histological lesions. For the other parameters analyzed, we observed that the consumption of lemon extract could improve the antioxidant activity in HPV16 mice. Further studies are needed to understand how the composition of the lemon juice extract influences the development of health status and treatment in this animal model and if different doses of this extract would cause some different effects.

## Figures and Tables

**Table 1:** Phenolic profile obtained by LC-DAD-ESI/MSn of fresh lemon juice with melatonin, expressed in mg 100 mL^−1^ (mean ± standard deviation).

Compound Name	^a^ Rt (min)	^b^ Molecular Ion ^c^ *(m/z)*	^c^ MS/MS (*m/z*)	^d^ λ max (nm)	Quantification
Cynarin	5	169	124, 78, 124	250,280	nd
Caffeic acid	6	463	300, 270, 300	250,280	1.5 ± 0.0
Gallic acid	8	609	301, 163, 150	288	nd
Diosmetin 6,8-di-*C*-glucoside (lucenin-2,4’-methyl ether)	10	624	608, 590, 530, 506, 488	250,268,342	13.7 ± 0.2
Diosmetin 8-*C*-glucoside (orientin 4’-methyl ether)	17	463	446, 428, 344, 314	250,268,342	12.9 ± 0.3
Eriocitrin (eriodictyol-7-*O*-rutinose)	19.1	179	135, 134, 106	285,325	19,5 ± 0.6
Luteolin-7-*O*-rutinoside	20.4	448	287, 153, 135	254,267	1.5 ± 0.2
Chrysoeriol 8-*C*-glucoside (scoparin)	22	462	446, 428, 314	255,268	12 ± 0.5
Apigenin 6,8-di-*C*-glucoside (vicenin-2)	23	594	560, 476, 458	268,334	11.5 ± 0.2
Hesperidin (hesperetin 7-*O*-rutinoside)	25	595	287, 150, 135	285,332	69.9 ± 3.9
Rutin	27	515	353, 191, 135	285,332	9.8 ± 0.6
Quercetin 3-*O*-glucoside	33.8	608	299, 270, 301	285,332	1.2 ± 0.0
					
Total phenolic content					153.5 ± 20.1

^a^ RT = retention time. ^b^ Molecular ion = tandem mass spectrometry. ^c^ MS/MS = molecular mass fragments. ^d^ λ max = wavelengths of maximum absorption in the visible region. nd = not detected.

**Table 2:** Evolution of bioactive compounds content and antioxidant capacity at room temperature (22 °C) at 24, 48 and 72 h in lemon juices treated with melatonin.

Functional Parameter	24 h	48 h	72 h	96 h
Vitamin C	32.04 ± 1.8	31.91 ± 2.1	29.22 ± 1.9	22.3 ± 1.6 ^1^
Total Phenolic Content	45.09 ± 3.2	43.96 ± 2.9	40.36 ± 3.3	33.14 ± 1.8 ^2^
Total Antioxidant Activity	24.63 ± 1.3	24.45 ± 1.6	21.16 ± 1.5	15.58 ± 1.5 ^3^

Data are the mean ± standard deviation (SD). Results of vitamin C have been expressed as mg 100 mL^−1^ of fresh weight (FW). Total phenolic content has been expressed as mg 100 g^−1^ of FW. TAA has been expressed as mg 100 g^−1^ of FW. ^1, 2, 3^. 96 h statistically different from 72 h (*p* < 0.05).

**Table 3:** Body weight variation in grams (mean ± SD) in drink test for each group ((G1, WT, control), (G2, WT, 1 mL lemon), (G3, WT, 1.5 mL lemon), (G4, WT, 2 mL), (G5, HPV16, control), (G6, HPV16, 1 mL lemon), (G6, HPV16, 1.5 mL lemon) and (G6, HPV16, 2 mL lemon)).

	G1 (WT, Control)	G2 (WT, 1 mL Lemon)	G3 (WT, 1.5 mL Lemon)	G4 (WT, 2 mL Lemon)	G5 (HPV16, Control)	G6 (HPV16, 1 mL Lemon)	G7 (HPV16, 1.5 mL Lemon)	G8 (HPV16, 2 mL Lemon)
1º sample date	28.05 ± 1.68	24.75 ± 1.91	28.76 ± 3.30	27.49 ± 1.79	27.39 ± 1.05	26.5 ± 1.67	26.88 ± 2.05	28.51 ± 1.09
2º sample date	26.9 ± 0.86 ^1^	22.38 ± 1.52 ^2^	26.69 ± 2.68	26.68 ± 1.81 ^3^	26.95 ± 1.85	23.41 ± 1.59	23.06 ± 3.02	26.41 ± 1.52
3º sample date	27.91 ± 0.86	24.67 ± 1.11	28.52 ± 2.94	27.91 ± 1.43	26.37 ± 1.28	26.07 ± 1.66	26.32 ± 2.54	28.41 ± 1.42
4ºsample date	27.92 ± 0.99	24.57 ± 0.74	27.93 ± 2.84	28.44 ± 1.8	19.87 ± 2.25	24.48 ± 1.89 ^4^	24.42 ± 2.14 ^5^	28.47 ± 1.24 ^6^
5º sample date	27.9 ± 1.85	24.81 ± 1.01	28.14 ± 3.09	28.98 ± 1.63	26.03 ± 1.44	26.39 ± 2.18	26.48 ± 2.05	28.7 ± 2.08
6º sample date	28.07 ± 0.84	24.34 ± 1.65	28.25 ± 2.73 ^7^	28.08 ± 1.84	26.7 ± 1.53	26.81 ± 1.47	27.4 ± 1.71	29.08 ± 1.44

^1^ G1 was statistically different from G2 (*p* < 0.05). ^2^ G2 was statistically different from G3 (*p* < 0.05). ^3^ G2 was statistically different from G4 (*p* < 0.05). ^4^ G6 was statistically different from G5 (*p* < 0.05). ^5^ G7 was statistically different from G5 (*p* < 0.05). ^6^ G8 was statistically different from G5 (*p* < 0.05). ^7^ G3 was statistically different from G2 (*p* < 0.05).

**Table 4:** Body weight variation in grams (mean ± SD) in food test for each group ((G1, WT, control), (G2, WT, 4 mL lemon), (G3, HPV16, control) and (G4, HPV16, 4 mL lemon)).

	G1 (WT, Control)	G2 (WT, 4 mL Lemon)	G3 (HPV16, Control)	G4 (HPV16, 4 mL Lemon)
1º sample date	29.87 ± 3.08	28.95 ± 1.59	26.96 ± 2.56	26.77 ± 2.66
2º sample date	32.03 ± 3.86	29.53 ± 1.25	28.38 ± 3.04	28.23 ± 2.56
3º sample date	29.72 ± 2.28	28.52 ± 0.69	29.02 ± 3.01	28.83 ± 2.72
4ºsample date	29.99 ± 2.44	28.01 ± 0.81	27.32 ± 2.10	27.09 ± 2.61
5º sample date	30.66 ± 2.46	27.94 ± 0.75	27.31 ± 2.50	27.52 ± 2.35
6º sample date	30.28 ± 2.40	28.45 ± 0.53	27.98 ± 2.15	28.51 ± 2.38

**Table 5:** Relative organ weights (grams) of the drinking test (mean ± SD).

	G1 (WT, Control)	G2 (WT, 1 mL Lemon)	G3 (WT, 1.5 mL Lemon)	G4 (WT, 2 mL Lemon)	G5 (HPV16, Control)	G6 (HPV16, 1 mL Lemon)	G7 (HPV16, 1.5 mL Lemon)	G8 (HPV16, 2 mL Lemon)
Spleen	0.0039 ± 0.0003 ^1^	0.0038 ± 0.0004	0.0042 ± 0.0004	0.0041 ± 0.0004	0.0051 ± 0.0005	0.0049 ± 0.0009	0.0046 ± 0.0007	0.0049 ± 0.0006
Heart	0.0041 ± 0.0003 ^2^	0.0046 ± 0.0007	0.0044 ± 0.0004	0.0045 ± 0.0004	0.0051 ± 0.0002	0.0048 ± 0.0005	0.0045 ± 0.0002	0.0045 ± 0.0005
Liver	0.0400 ± 0.0022	0.0419 ± 0.0030	0.0417 ± 0.0017 ^3^	0.0426 ± 0.0017	0.0473 ± 0.0010	0.0461 ± 0.0028	0.0468 ± 0.0030	0.0470 ± 0.0010
Lung	0.0078 ± 0.0009	0.0073 ± 0.0002	0.0067 ± 0.0004	0.0071 ± 0.0003	0.0080 ± 0.0009	0.0068 ± 0.0004	0.0080 ± 0.0026	0.0072 ± 0.0004
Kidney (left)	0.0054 ± 0.0008	0.0058 ± 0.0005	0.0054 ± 0.0008	0.0050 ± 0.0006	0.0065 ± 0.0002	0.0057 ± 0.0004	0.0060 ± 0.0008	0.0061 ± 0.0004
Kidney (right)	0.0054 ± 0.0005	0.0055 ± 0.0007	0.0054 ± 0.0009	0.0057 ± 0.0007	0.0062 ± 0.0003	0.0054 ± 0.0007	0.0058 ± 0.0002	0.0053 ± 0.0003

^1^ Statistically different from G5 (*p* < 0.05). ^2^ Statistically different from G5 (*p* < 0.05). ^3^ Statistically different from G7 (*p* < 0.05).

**Table 6:** Relative organ weights (grams) of the diet assay (mean ± SD).

	G1 (WT, Control)	G2 (WT, 4 mL Lemon)	G3 (HPV16, Control)	G4 (HPV16, 4 mL Lemon)
Spleen	0.0044 ± 0.0009	0.0047 ± 0.0005	0.0050 ± 0.0008	0.0057 ± 0.0015
Heart	0.0049 ± 0.0007	0.0058 ± 0.0009	0.0048 ± 0.0004	0.0048 ± 0.0009
Liver	0.0466 ± 0.0034	0.0495 ± 0.0049	0.0482 ± 0.0053	0.0478 ± 0.0045
Lung	0.0081 ± 0.0005	0.0081 ± 0.0008	0.0069 ± 0.0008	0.0069 ± 0.0008
Kidney (left)	0.0059 ± 0.0006	0.0060 ± 0.0003	0.0055 ± 0.0014	0.0063 ± 0.0006
Kidney (right)	0.0067 ± 0.0012	0.0061 ± 0.0005	0.0061 ± 0.0011	0.0056 ± 0.0006

**Table 7:** Microhematocrit (Ht) and PPT (mean ± SD) values for the drink assay for each group.

Groups	G1 (WT, Control)	G2 (WT, 1 mL Lemon)	G3 (WT, 1.5 mL Lemon)	G4 (WT, 2 mL lemon)	G5 (HPV16, Control)	G6 (HPV16, 1 mL Lemon)	G7 (HPV16, 1.5 mL Lemon)	G8 (HPV16, 2 mL Lemon)
Ht (%)	46.5	45.6	46.34	45.62	43.76	44.55	45.42	45.04
PPT (g/dl)	4.78 ± 0.25	4.56 ± 0.09	4.94 ± 0.22	4.98 ± 0.28	5.1 ± 0.2	5 ± 0.12	5.32 ± 0.28	5.18 ± 0.53

**Table 8:** Microhematocrit (Ht) and PPT (mean ± SD) values for the drink assay for each group.

Groups	G1 (WT, Control)	G2 (WT, 4 mL Lemon)	G3 (HPV16, Control)	G4 (HPV16, 4 mL Lemon)
Ht (%)	47.98	48.38	45.8	47.9
PPT (g/dl)	4.95 ± 0.24	5.02 ± 0.39	5.78 ± 0.46	5.36 ± 0.18

**Table 9:** Number of animals (%) with histological lesions in all experimental groups of drink test.

Organs	Lesion	G1 (WT, Control)	G2 (WT, 1 mL Lemon)	G3 (WT, 1.5 mL Lemon)	G4 (WT, 2 mL Lemon)	G5 (HPV16, Control)	G6 (HPV16, 1 mL Lemon)	G7 (HPV16, 1.5 mL Lemon)	G8 (HPV16, 2 mL Lemon)
Liver	Normal	2/5 (40%)	5/5 (100%)	3/5 (60%)	3/5 (60%)	3/5 (60%)	2/5 (40%)	2/5 (40%)	3/5 (60%)
	Hepatitis	3/5 (60%)	0/5 (0%)	2/5 (40%)	2/5 (40%)	2/5 (40%)	2/5 (40%)	3/5 (60%)	2/5 (40%)
Spleen	Normal	5/5 (100%)	5/5 (100%)	5/5 (100%)	5/5 (100%)	5/5 (100%)	5/5 (100%)	5/5 (100%)	5/5 (100%)
Kidney	Normal	4/5 (80%)	5/5 (100%)	3/5 (60%)	2/5 (40%)	1/5 (20%)	4/4 (100%)	4/5 (80%)	4/5 (80%)
	Nephritis	1/5 (20%)	0/5 (0%)	2/5 (40%)	3/5 (60%)	4/5 (80%)	0/4 (0%)	1/5 (20%)	1/5 (20%)
Lung	Normal	5/5 (100%)	5/5 (100%)	5/5 (100%)	5/5 (100%)	5/5 (100%)	5/5 (100%)	5/5 (100%)	5/5 (100%)
Chest skin	Normal	5/5 ^1^ (100%)	5/5 (100%)	5/5 ^2^ (100%)	5/5 ^3^ (100%)	0/5 (0%)	0/4 (0%)	0/5 (0%)	0/5 (0%)
	Hyper- plasia	0/5 (0%)	0/5 (0%)	0/5 ^4^ (0%)	0/5 (0%)	2/5 (40%)	3/4 (75%)	5/5 (100%)	4/5 (80%)
	Dysplasia	0/5 (0%)	0/5 (0%)	0/5 (0%)	0/5 (0%)	3/5 (60%)	1/4 (25%)	0/5 (0%)	1/5 (20%)
	SCC	0/5 (0%)	0/5 (0%)	0/5 (0%)	0/5 (0%)	0/5 (0%)	0/4 (0%)	0/5 (0%)	0/5 (0%)
Ear skin	Normal	5/5 ^5^ (100%)	5/5 (100%)	5/5 ^6^ (100%)	5/5 ^7^ (100%)	0/5 (0%)	0/4 (0%)	0/5 (0%)	0/5 (0%)
	Hyper- plasia	0/5 (0%)	0/5 (0%)	0/5 (0%)	0/5 (0%)	0/5 (0%)	1/4 (25%)	1/5 (25%)	2/5 (40%)
	Dysplasia	0/5 (0%)	0/5 (0%)	0/5 (0%)	0/5 (0%)	4/5 (80%)	3/4 (75%)	4/5 (80%)	3/5 (60%)
	SCC	0/5 (0%)	0/5 (0%)	0/5 (0%)	0/5 (0%)	1/5 (20%)	0/4 (0%)	0/5 (0%)	0/5 (0%)

^1^ Significantly different from G5 (*p* < 0.05). ^2^ Significantly different from G7 (*p* < 0.05). ^3^ Significantly different from G8 (*p* < 0.05). ^4^ Significantly different from G7 (*p* < 0.05). ^5^ Significantly different from G5 (*p* < 0.05). ^6^ Significantly different from G7 (*p* < 0.05). ^7^ Significantly different from G8 (*p* < 0.05).

**Table 10:** Number of animals (%) with histological lesions in all experimental groups of diet test.

Organs	Lesion	G1 (WT, Control)	G2 (WT, 4 mL Lemon)	G3 (HPV16, Control)	G4 (HPV16, 4 mL Lemon)
Liver	Normal	5/5 (100%)	5/5 (100%)	5/5 (100%)	5/5 (100%)
	Hepatitis	0/5 (0%)	0/5 (0%)	0/5 (0%)	0/5 (0%)
Spleen	Normal	5/5 (100%)	5/5 (100%)	5/5 (100%)	5/5 (100%)
Kidney	Normal	5/5 (100%)	2/5 (20%)	4/5 (80%)	4/5 (80%)
	Nephritis	0/5 (0%)	3/5 (60%)	1/5 (20%)	1/5 (20%)
Lung	Normal	5/5 (100%)	5/5 (100%)	5/5 (100%)	5/5 (100%)
Chest skin	Normal	5/5 ^1^ (100%)	5/5 ^2^ (100%)	0/5 (0%)	0/5 (0%)
	Hyperplasia	0/5 (0%)	0/5 (0%)	4/5 (80%)	4/5 (80%)
	Dysplasia	0/5 (0%)	0/5 (0%)	1/5 (20%)	1/5 (20%)
	SCC	0/5 (0%)	0/5 (0%)	0/5 (0%)	0/5 (0%)
Ear skin	Normal	5/5 ^3^ (100%)	5/5 ^4^ (100%)	0/5 (0%)	0/5 (0%)
	Hyperplasia	0/5 (0%)	0/5 (0%)	1/5 (20%)	0/5 (0%)
	Dysplasia	0/5 (0%)	0/5 (0%)	3/5 (60%)	5/5 (100%)
	SCC	0/5 (0%)	0/5 (0%)	1/5 (20%)	0/5 (0%)

^1^ Significantly different from G3 (*p* < 0.05). ^2^ Significantly different from G4 (*p* < 0.05). ^3^ Significantly different from G3 (*p* < 0.05). ^4^ Significantly different from G4 (*p* < 0.05).

**Table 11:** Oxidative stress parameters evaluated in the liver of the drink test mice (mean ± SD).

Groups	G1 (WT, Control)	G2 (WT, 1 mL Lemon)	G3 (WT, 1.5 mL Lemon)	G4 (WT, 2 mL Lemon)	G5 (HPV16, Control)	G6 (HPV16, 1 mL Lemon)	G7 (HPV16, 1.5 mL Lemon)	G8 (HPV16, 2 mL Lemon)
SOD	20.75 ± 3.03	27.31 ± 7.93	27.21 ± 3.1 ^1^	42.87 ± 7.67 ^2,3^	35.21 ± 6.52	19.34 ± 4.98 ^6,7^	27.18 ± 1.63 ^8^	26.74 ± 3.52
CAT	9.35 ± 2.23	11.51 ± 1.36	12.06 ± 0.85	14.69 ± 1.07 ^4,5^	13.94 ± 1.43	7.65 ± 1.03 ^9,10^	10.14 ± 1.55 ^11^	11.49 ± 2.16

Data are the mean ± DS of five independent experiments performed in duplicate. Results of SOD activity have been expressed as U min^−1^ mg^−1^. CAT activity content has been expressed as mmol H_2_O_2_ min^−1^mg^−1^). ^1,2^ G1 was significantly different from G3 and G4 (*p* < 0.05). ^3^ G4 was significantly different from G2 and G3 (*p* < 0.05). ^4^ G4 was significantly different from the G1 (*p* < 0.05). ^5^ G4 was significantly different from G2 and G3 (*p* < 0.05). ^6,8,9,11^ G5 was significantly different from G6 and G7 (*p* < 0.05). ^7,10^ G6 was significantly different from G7 and G8 (*p* < 0.05).

**Table 12:** Oxidative stress parameters evaluated in the liver of diet test mice (mean ± SD).

Groups	G1 (WT, Control)	G2 (WT, 4 mL Lemon)	G3 (HPV16, Control)	G4 (HPV16, 4 mL Lemon)
SOD	27.81 ± 6.04	28.24 ± 4.67	35.66 ± 0.29	33.58 ± 2.58
CAT	7.65 ± 1.54	8.26 ± 1.02	12.68 ± 0.91	13.29 ± 1.17

Data are the mean ± DS of five independent experiments performed in duplicate. Results of SOD activity have been expressed as U min^−1^ mg^−1^. CAT activity content has been expressed as mmol H_2_O_2_ min^−1^mg^−1^).

## Data Availability

Data is contained within the article.
